# Examining the Relationship between Choice, Therapeutic Alliance and Outcomes in Mental Health Services

**DOI:** 10.3390/jpm3030191

**Published:** 2013-08-20

**Authors:** Victoria Stanhope, Stacey L. Barrenger, Mark S. Salzer, Stephen C. Marcus

**Affiliations:** 1 Silver School of Social Work, New York University, 1 Washington Square N, New York, NY 10003, USA; E-Mail: Stacey.barrenger@nyu.edu; 2 Department of Rehabilitation, Temple University, 1700 N. Broad Street, Suite 304, Philadelphia, PA 19122, USA; E-Mail: msalzer@temple.edu; 3 School of Social Policy and Practice, Caster Building, University of Pennsylvania, Philadelphia, PA 19104, USA; E-Mail: marcuss@sp2.upenn.edu

**Keywords:** self-determination, therapeutic alliance, choice, mental health services

## Abstract

**Background:** Self-determination within mental health services is increasingly recognized as an ethical imperative, but we still know little about the impact of choice on outcomes among people with severe mental illnesses. This study examines whether choice predicts outcomes and whether this relationship is mediated by therapeutic alliance. **Method:** The study sample of 396 participants completed a survey measuring choice, therapeutic alliance, recovery, quality of life and functioning. Multivariate analyses examined choice as a predictor of outcomes, and Sobel tests assessed alliance as a mediator. **Results:** Choice variables predicted recovery, quality of life and perceived outcomes. Sobel tests indicated that the relationship between choice and outcome variables was mediated by therapeutic alliance. **Implications:** The study demonstrates that providing more choice and opportunities for collaboration within services does improve consumer outcomes. The results also show that collaboration is dependent on the quality of the relationship between the provider and consumer.

## 1. Introduction

In providing services, users’ choice about their treatment is a key aspect of healthcare reform in the 21st century. Driving this transformation within mental healthcare is the concept of recovery, which posits that all people with severe mental illnesses can pursue meaningful lives and have a right to services that are oriented towards their individualized goals [1]. Moreover, a less emphasized, but nevertheless important, aspect of evidence-based practice is balancing patient preference and values with empirically supported treatments, underscoring how choice must be integral to all aspects of service delivery. Choice is essentially self-determination, which can be understood as a right, “by the very essence of being human, people have the right to choose where they want to live, what they want to work at, with whom they wish to affiliate and how they wish to enjoy spiritual, educational and recreational pursuits” (ref. [2] page 170). Choice has also been identified as a fundamental human capability [3], which in mental health settings means varying approaches depending on individual preference rather than prescribing based on the provider perspective [4]. To support the shift to service user choice within mental healthcare, the National Institute of Mental Health [5] is now calling for research on “personalization”, which is the matching of interventions to individual characteristics and preferences. A recent meeting of stakeholders on disparities characterized interventions that facilitate choice and shared decision making as “low hanging fruit,” meaning they can have an immediate impact on improving adherence [6].

Whether people both perceive and actually have choices when they are receiving services is often determined by their interactions with providers. Historically, the interactions between physician and service users have consisted of physicians taking the leading role in information exchange, deliberation and decision making, with service users being largely passive in the treatment process [7]. Patient centeredness is one approach that seeks to shift this dynamic by having doctors understand the person holistically, share power and responsibility for decisions and build a therapeutic alliance [8]. Shared decision making is another mechanism used to promote choice through positioning the service user as the expert in their recovery and the doctor providing information, discussing options and, ultimately, supporting service user decisions [9].

Self-determination can be promoted throughout mental health services, resulting in consumers having more choice about housing, therapy, group participation, employment or community integration. Within psychiatry, self-determination has mostly been understood in relation to medication management. There is a growing body of evidence both in healthcare and mental healthcare suggesting that increasing choice promotes positive outcomes [10]. One study found that when patients were better informed of the risks and benefits of antipsychotic medication, 87% chose to adhere to the medication [11]. Another study found when clients received therapy that integrated shared decision making, they had greater adherence to medications [12]. Shared decision making has also been associated with increased satisfaction with services and improved social functioning among people with schizophrenia [13]. However, there is a need for additional research on how different groups prioritize health states, how environmental factors influence health behaviors and choices and how much choice consumers wish to exercise [14]. Furthermore, needed are ways to better operationalize choice, engagement and adherence, in addition to examining how these factors operate in the real world [10].

Patient centeredness and shared decision making have been widely embraced as practice principles within health and mental health services [15,16,17], which necessitates a better understanding of the conditions that promote an active service user role and meaningful collaboration. Particularly within a mental health system that has often disempowered consumers, due to stigma and paternalistic attitudes, service users may not feel comfortable or trusting enough to engage on a more equal footing with their provider. One important component in service encounters is the therapeutic alliance, which speaks to the trust and comfort level between the service user and the provider. This construct has been referred to as therapeutic alliance, working relationship or working alliance in the literature and can encompass the relationship between the service user and various providers, including therapists, case managers and psychiatrists. Important components of the therapeutic alliance are service user and provider agreement on goals, service user and provider agreement on tasks and the bond between the service user and the provider [18]. In the psychotherapy literature, there is strong, consistent evidence demonstrating a positive relationship between therapeutic alliance and outcomes [19].

Mental health services research has examined the therapeutic alliance, mostly in the context of the service user-case manager dyad, finding support for an association between therapeutic alliance and retention in treatment, reduced symptomology, improved social functioning and employment [20]. In an examination of the meaning of the therapeutic alliance within community support programs, Kirsh and Tate [21] found offering choices to be an integral part of building alliances and negotiating medication use. Both service users and providers understood choice to include the process of laying out options for service users, rather than “pushing” medication. Likewise, having input into treatment, feeling known and being allowed to talk were identified by service users as key components of their relationships with providers in addition to receiving instrumental help [22]. In a longitudinal study testing the effectiveness of a medication alliance intervention for clinicians, Bryne and Deane [23] found that insight gained through the working alliance accounted for increased medication adherence and improved outcomes for service users. Weiss and colleagues [24] showed that working alliance was not only strongly associated with medication adherence over time, but was also strongly related to service users becoming medication-adherent when they had not been previously. 

Although several have suggested the utility of the therapeutic alliance in the negotiations between psychiatrists and service users about medication [25,26], few studies have examined the effects of the service user-psychiatrist relationship on outcomes. In a review of health communication, Cruz and Pincus [27] found that therapeutic alliance with a psychiatrist was associated with outcomes, such as patient satisfaction, adherence to pharmacological treatment and keeping appointments. Strauss and Johnson [28] found that stronger alliances between service users and psychiatrists predicted fewer manic symptoms, less negative attitudes about medication and less stigma about bipolar disorder. Furthermore, provider behaviors, such as “*conveying confidence*” in consumers’ ability to participate in treatment, “*staying in regular contact*” and “*regularly reviewing progress*” with service users contributed to increased medication adherence [29]. While the direct association between therapeutic alliance and outcomes has been more commonly studied, Guadiano and Miller [30] examined if the therapeutic alliance mediated the relationship between service users’ expectations about psychiatric improvement following an inpatient stay and adherence and symptoms. They found strong evidence for the mediational effects of both psychiatrist and service user ratings of alliance on the relationship between expectations and time in treatment. In considering shared decision making and medication management, Deegan and Drake [15] have called for more research exploring decisional dynamics in psychiatric encounters. 

Our study seeks to contribute to the evidence base by investigating how choice impacts service user outcomes and the role of therapeutic alliance in mediating this relationship. Based on a large dataset of people with severe mental illnesses receiving services at four different mental health clinics, the study addresses the following questions: (1) To what extent does service user choice predict outcomes; (2) Does therapeutic alliance mediate the effect of service user choice on outcomes? 

## 2. Experimental

### 2.1. Participants and Procedures

The cross-sectional study was a secondary data analysis using data from a study related to mental health disparities, which was conducted from 2004 to 2007. The disparities study consecutively recruited 396 participants who were enrolled in services at four community mental health clinics. Inclusion criteria were: a primary diagnosis of schizophrenia spectrum disorder or major depressive disorder; currently receiving psychiatric medication prescriptions from the community mental health clinic; having the racial identity of white or African American; and being over the age of 18. Participants completed structured interviews, which focused on service use, treatment process and outcomes. The data source for this study was the baseline interview for the disparities study, which was completed by all 396 participants. Institutional Review Board approval from the University of Pennsylvania was obtained for the study.

### 2.2. Measures

Gender, race, diagnosis and length of time in services and with psychiatrist were measured by demographic and clinical questions from the baseline interview. Severity of illness was measured by the Colorado Symptom Index (CSI) [31], which is specifically used to assess symptoms related to mania and psychosis. The CSI has demonstrated good reliability and validity [32]. The scale has 10 items, with responses ranging from 1 to 5. Scale scores were based on an averaging of all items, with total scores ranging from 1 to 5, with higher scores representing a greater severity of illness.

Choice was operationalized as patient perception of patient centeredness and patient perception of consultation tasks. Patient perception of patient centeredness was measured by the Patient Perception of Patient Centeredness Scale [33]. The validity of the 14-item scale was established through significant correlations with patient health outcomes. The scale has a reliability of 0.71 [34]. The scale has 14 items, with responses ranging from 1 to 4. Scale scores were based on averaging all items, with total scores ranging from 1 to 4, with higher scores representing greater perception of patient centeredness. Patient perception of consultation tasks was measured by the Patient Perception of Consultation Tasks Scale, which was developed for the disparities study. The seven questions ask specific questions about whether the psychiatrist gave responsibility for making decisions to the services user, whether he or she gave the information necessary to make decisions about medication and whether he and she asked for the service user’s opinion about taking the medication that had been prescribed. The scale had a Cronbach’s alpha of 0.78 and was correlated with the Patient Perception of Patient Centeredness skills measure (r = 0.47, *p* < 0.01). The scale has 7 items, with responses ranging from 1 to 6. Scale scores were based on averaging all items, with total scores ranging from 1 to 6, with higher scores representing greater perceived consultation.

Therapeutic alliance was used to measure the service user-psychiatrist relationship from the service user perspective. The Working Alliance Inventory-Short Version (WAI-S) is based upon Bordin’s [18] three factor conceptualization of the provider and client relationship: collaboration on tasks, collaboration on goals and the bond between the client and therapist. Coefficient alphas for the WAI-S three subscales have ranged from 0.85 to 0.92 [35]. The WAI-S was highly correlated to the California Psychotherapy Alliance Scale (r = 0.80) and the Penn Helping Alliance Interview Schedule (r = 0.74) [36]. For this study, the WAI-S had good internal consistency (α = 0.88). The scale has 12 items, with responses ranging from 1 to 7. Scale scores were based on averaging all items, with total scores ranging from 1 to 7, with higher scores representing a more positive working alliance. 

Outcomes were operationalized as recovery, quality of life and perceived outcomes. Recovery was measured by the Recovery Assessment Scale—Short Form, which assesses the subjective aspects of recovery. The scale has established reliability and validity [37]. The scale has 20 items, with responses ranging from 1 to 5. Scale scores were based on averaging all items, with total scores ranging from 1 to 5, with higher scores representing greater recovery. Quality of life was measured by the short form of the Quality of Life Interview subjective component [38], including questions about family, social life and living situation. The scale has demonstrated satisfactory validity and reliability [39]. The scale has 6 items, with responses ranging from 1 to 7 (terrible to delighted). Scale scores were based on an averaging of all items, with total scores ranging from 1 to 7, with higher scores representing greater quality of life. Perceived outcomes were measured by the Mental Health Statistical Improvement Program (MHSIP) outcomes scale [40], which has questions relating to dealing more effectively with mental health, social relationships and living independently. This instrument has good evidence of reliability and validity [41]. The scale has 14 items, with responses ranging from 1 to 5. Scale scores were based on an averaging of all items, with total scores ranging from 1 to 5, with higher scores representing better perceived outcomes. 

### 2.3. Analysis

Univariate analyses were conducted to generate frequencies for gender, race and diagnosis. Means, range and standard deviations were calculated for each of the measures and length of time in services with psychiatrist. Multivariate analysis examined choice as a predictor of outcomes, controlling for gender, race, diagnosis, severity of illness and length of time in services with psychiatrist. Three models tested the extent to which perception of patient centeredness and perception of consultation tasks predicted recovery, quality of life and perceived outcomes. R^2^ was calculated to estimate the amount of variance explained by study variables on outcomes. All models present standardized beta coefficients. We conducted bivariate analysis to examine the direct relationships between choice and working alliance and working alliance and outcomes. Sobel tests were then conducted to assess whether therapeutic alliance mediated any of the relationships between each of the three choice variables and each of the three outcomes.

## 3. Results

In the sample of 396, 59% were female and 62% were African-American (see Table 1). The majority of the sample was diagnosed with schizophrenia (60%), and the remaining participants were diagnosed with major depression. The average length participants had received services from the agency was 8.2 years, and the average time participants had seen their psychiatrist was 3.2 years.

**Table 1:** Demographics, treatment process and outcomes.

		N	%
		396	
Gender			
	Male	163	41
	Female	233	59
Race			
	African American	247	62
	Caucasian	149	38
Diagnosis			
	Schizophrenia	238	60
	Depression	158	40
		**Mean**	**SD**
Length of Time in Services (years)			
	Receiving services at clinic	8.2	7
	Receiving services from psychatrist	3.2	4.4
Severity of Illness			
	Colorado Symptom Index	2.3	1
Treatment Process			
	Working Alliance Inventory	5.4	1.2
	Patient Consultation	4.2	0.7
	Patient Centeredness	3.3	0.6
Outcomes			
	Recovery	3.9	0.5
	Quality of Life	4.6	0.9
	Perceived Outcomes	3.9	0.7

The regression analyses demonstrated that service user choice had a strong association with service user outcomes (see Table 2), when controlling for race, gender and diagnosis. Perception of consultation tasks predicted greater recovery (β = 0.312, *p* < 0.01), higher quality of life (β = 0.129, *p* < 0.01) and improved perceived outcomes (β = 0.309, *p* < 0.01). Perception of patient centeredness predicted greater recovery (β = 0.144, *p* < 0.01), higher quality of life (β = 0.116, *p* < 0.01) and improved perceived outcomes (β = 0.204, *p* < 0.01). The R^2^ for these models ranged from 0.16 to 0.32, demonstrating that choice, together with significant control variables, played a meaningful role in the variance among the outcome variables. Significant control variables were severity of illness, race and diagnosis (see Table 2). Severity of illness predicted lower rates of recovery, less quality of life and poorer perceived outcomes. For race, African Americans reported higher levels of recovery than whites, and for diagnosis, people diagnosed with schizophrenia reported higher levels of recovery, but worse perceived outcomes than people with major depression. 

**Table 2:** Regression analyses examining the relationship between choice and outcomes.

Choice	Outcomes		
	Recovery	Quality of Life	Perceived Outcomes
	β	β	β
**Perception of Consultation**	.312**	.129*	.309**
**Perception of Patient Centeredness**	.144**	.116*	.204**
**Severity of Illness**	-.26**	-.270**	-.253**
**Female**	NS	NS	NS
**African American**	.103*	NS	NS
**Schizophrenia**	.147**	NS	-.139**
**Length of Time in Services with Psychiatrist**	NS	NS	NS
**R^2^**	.31	.16	.32

* - significant at .05 level; ** - significant at .005 Level; NS - not significant.

Bivariate analyses and Sobel tests were conducted to answer the second study question, whether therapeutic alliance mediated any of the relationships between choice and outcomes. Bivariate analyses examining the relationships between choice variables and working alliance and working alliance and outcome variables found strong associations between all the variables (see Table 3). Correlations ranged between 0.215 and 0.698, and all were significant (*p* < 0.01). Sobel tests examined whether working alliance mediated the relationships between the choice and outcome variables when controlling for gender, race diagnosis, severity of illness and length of time in services with psychiatrist. Working alliance was found to mediate the relationships between: perception of patient centeredness and recovery (z = 3.16, *p* < 0.01); patient perception of patient centeredness and perceived outcomes (z = 2.99, *p* < 0.01); perception of patient consultation and recovery (z = 3.4, *p* < 0.01); perception of patient consultation and quality of life (z = 2.16, *p* = 0.03); and perception of patient consultation and perceived outcomes (z = 3.87, *p* < 0.01). The only mediation found not to be significant was between perception of patient centeredness and quality of life, indicating that the positive effect of perception of patient centeredness on quality of life was not mediated by working alliance. 

**Table 3:** Zero order correlations between choice, working alliance and outcomes.

	1	2	3	4	5	6
1. Working Alliance Inventory	1	.450**	.698**	.362**	.238**	.403**
2. Perception of Consultation		1	.489**	.423**	.231**	.440**
3. Perception of Patient Centeredness			1	.330**	.215**	.392**
4. Recovery				1	.496**	.598**
5. Quality of Life					1	.438**
6. Perceived Outcomes						1

* - significant at .05 level; ** - significant at .005 Level.

## 4. Discussion

The study supports the notion that providing people with more choice predicts better outcomes for people with severe mental illnesses. The finding that perceiving one’s psychiatrist to be patientcentered and consultative was positively related to outcomes confirms that, beyond the ethical imperative, there is an empirical basis for embedding self-determination within services. These findings counter some of the concerns that recovery-oriented services can, in fact, be detrimental for people with severe mental illnesses by allowing them to make decisions that may be harmful [42]. The justification for coercive treatment has been predicated on a belief that people with severe mental illnesses lack insight and, therefore, are not capable of making decisions about their treatment [43]. This study adds to the growing evidence that not only can consumers make decisions that promote their recovery, but are more likely to recover if they are offered the opportunity to make choices about their treatment. Moreover, choice not only had a robust relationship with the more subjective elements of recovery, such as feeling hopeful, but also with more objective indicators, such as being able to cope with daily problems and make progress towards one’s goals. These relationships were found while controlling for severity of illness, indicating that choice plays a positive role in people’s recovery, regardless of symptomology.

Recovery narratives have consistently underscored the role of self-determination in realizing a sense of personhood [44]. These results demonstrate that attending to self-determination within services by offering service users greater choice and input into their treatment sustains beyond the treatment experience to impact longer term outcomes. Self-determination theory explains how promoting internal motivation by allowing people to be curious, explore and show mastery can, in turn, lead to external motivation, which is acting to attain goals [45]. By understanding the critical role that choice plays in motivation and goal attainment, we can more effectively address the persistent problem of service disengagement by focusing on the mutable aspects of the treatment process. 

However, recovery remains a complex phenomenon, and this was reflected in our findings indicating differential outcomes among subsets of participants. Symptom severity has often been conceptualized as a more objective aspect of recovery, whether measured by self-report or by a clinician. In this study, higher levels of self-reported symptoms had a negative relationship with the more subjective aspects of recovery, as well as quality of life and perceived outcomes. This adds to already mixed findings, with some studies finding a relationship between objective and subjective aspects of recovery, while others find none [46,47]. There were differences according to diagnosis, with people with schizophrenia reporting higher rates of recovery than people with major depression, but rating lower on perceived outcomes. As a measure of daily functioning, perceived outcomes, although based on self-report, can also be viewed as a more objective aspect of recovery. The findings suggest that for people with schizophrenia, activities, such as work, may not be as central to their sense of recovery. In addition, African Americans reported higher rates of recovery than whites. These findings all speak to the complex question of what constitutes recovery and how much one’s perception of recovery is related to expectations, the nature and course of one’s illness and the quality of one’s life prior to diagnosis.

The study also found that therapeutic alliance plays an important role in the impact of choice on outcomes. This finding gives some context to the treatment processes that accompany offering service users’ choices and implementing shared decision making. Drake and Deegan [15] describe the negotiation process between psychiatrists and service users over medication management as a potential area for conflict. This study indicates that therapeutic alliance can play a helpful role in that dynamic—indeed, when psychiatrists offer choices and consult with service users, this is far more effective when there is a strong working alliance. Even when being offered choices, consumers may be reluctant to enter into a collaborative relationship with a provider they do not like or do not trust. This suggests that some groundwork must be done in terms of relationship building to facilitate shared decision making, even within the constraints of short psychiatric visits. Research has also shown that even objective coercion is perceived very differently, depending on the quality of the interaction and the therapeutic alliance [48,49]. Therefore, decisional conflicts or disagreement could be understood differently and have different outcomes, if they occur within the context of a trusting relationship between the service user and psychiatrist. Overall, this finding indicates that similar to other mental health services, the quality of the relationship plays an important role in determining outcomes within mediation management. 

There are some limitations to the study. The study was cross-sectional, and therefore, we cannot draw causal conclusions. We were unable to control for other services that participants may have been receiving, and therefore, the improvement in outcomes may have been due to other services, rather than greater choice in psychiatric treatment. Finally, most participants were actively involved in services and had a history of service engagement; therefore, service users who are prone to disengage from services and who may perceive choice and relationships differently were not included in the study.

## 5. Conclusions

Empirical support for the notion that giving people choices within mental health services leads to positive outcomes is vital as agencies take steps to transform their services according to the principles of recovery. Agencies now need guidance about specific practices, such as person-centered care planning and shared decision making, which will enhance choice within their programs and improve the collaborative dynamics between service users and providers. Any concerns about relinquishing power by the mental health professions, such as worries about liability and risk [50], can be eased by evidence that service users benefit from having greater choice in their own care. Specifically, training for psychiatrists needs to reflect this new emphasis on choice and shared decision making. This training can be informed by research on the therapeutic alliance, and that enacting choice is more effective when it occurs in the context of a trusting relationship. The brevity and focus of psychiatric visits on medication use only presents a potential structural barrier for recovery-oriented practice within psychiatry and needs to be addressed with system level reform. As behavioral health moves to performance-based reimbursement models, this may allow more flexibility in structuring psychiatry encounters that can genuinely promote choice for service users and lead to meaningful outcomes for people with severe mental illnesses. 
